# Anomalous Gray Matter Structural Networks in Patients with Hepatitis B Virus-Related Cirrhosis without Overt Hepatic Encephalopathy

**DOI:** 10.1371/journal.pone.0119339

**Published:** 2015-03-18

**Authors:** Xiao-Fei Lv, Kai Liu, Ying-Wei Qiu, Pei-Qiang Cai, Jing Li, Gui-Hua Jiang, Yan-Jia Deng, Xue-Lin Zhang, Pei-Hong Wu, Chuan-Miao Xie, Ge Wen

**Affiliations:** 1 Imaging and Interventional Radiology, Sun Yat-sen University Cancer Center; State Key Laboratory of Oncology in South China; Collaborative Innovation Center for Cancer Medicine, Guangzhou, People's Republic of China; 2 Medical Imaging Centre, Nanfang Hospital, Southern Medial University, Guangzhou, People’s Republic of China; 3 Department of medical imaging, The First Affiliated Hospital of Gannan Medical University, Ganzhou, People's Republic of China; 4 Department of Medical Imaging, Guangdong No. 2 Provincial People’s Hospital, Guangzhou, People's Republic of China; Wake Forest School of Medicine, UNITED STATES

## Abstract

**Background and Purpose:**

Increasing evidence suggests that cirrhosis may affect the connectivity among different brain regions in patients before overt hepatic encephalopathy (OHE) occurs. However, there has been no study investigating the structural reorganization of these altered connections at the network level. The primary focus of this study was to investigate the abnormal topological organization of the structural network in patients with hepatitis B virus-related cirrhosis (HBV-RC) without OHE using structural MRI.

**Methods:**

Using graph theoretical analysis, we compared the global and regional topological properties of gray matter structural networks between 28 patients with HBV-RC without OHE and 30 age-, sex- and education-matched healthy controls. The structural correlation networks were constructed for the two groups based on measures of gray matter volume.

**Results:**

The brain network of the HBV-RC group exhibited a significant decrease in the clustering coefficient and reduced small-worldness at the global level across a range of network densities. Regionally, brain areas with altered nodal degree/betweenness centrality were observed predominantly in association cortices (frontal and temporal regions) (p < 0.05, uncorrected), including a significantly decreased nodal degree in the inferior temporal gyrus (p < 0.001, uncorrected). Furthermore, the HBV-RC group exhibited a loss of association hubs and the emergence of an increased number of non-association hubs compared with the healthy controls.

**Conclusion:**

The results of this large-scale gray matter structural network study suggest reduced topological organization efficiency in patients with HBV-RC without OHE. Our findings provide new insight concerning the mechanisms of neurobiological reorganization in the HBV-RC brain from a network perspective.

## Introduction

An estimated 350 million people suffer from chronic hepatitis B viral infection worldwide, and more than 75% of these chronically infected people live in Asia [1]. Annually, approximately 1.0–2.4% of these patients progress to hepatitis B virus-related cirrhosis (HBV-RC) [1]. Hepatic encephalopathy (HE) is one of the most common complications of end-stage cirrhosis [1]. This condition is characterized by a wide range of neuropsychiatric abnormalities that can lead to coma and death [2]. Clinically, overt hepatic encephalopathy (OHE) can be readily identified based on the presence of neurological manifestations that are obvious upon clinical examination [3]. However, cirrhotic patients commonly present with a spectrum of neuropsychological symptoms, even without any clinical symptoms of OHE. Neuropsychological and neurophysiological testing have revealed various degrees of cognitive deficits [4,5] in cirrhotic patients before OHE occurs, which may lead to a poorer quality of life, deterioration in daily functioning, and even increased morbidity [3].

Over the past decade, various neuroimaging studies [6–13] have been proposed to explore the pathophysiological mechanisms of the cognitive abnormalities observed in cirrhotic patients without OHE. Previous studies from our lab and others [6–8,13] have used resting-state functional MRI to identify cirrhosis-related abnormalities not only in localized brain regions but also in the functional connections among a series of brain areas. Even the functional integration of some specific sub-networks (such as the dorsal attention network, the default network, the auditory network and the visual network) [9,13] and the whole-brain network [8,10] has been implicated. Thus, a wide range of cirrhosis-related cognitive deficits may arise from disturbed connectivity among a series of brain regions rather than segregated regional brain abnormalities.

Additionally, neuroanatomical studies, such as MRI-based brain structure analyses, have also been used to investigate the etiopathogenesis of impaired cognition in cirrhotic patients [11–17]. Voxel-based morphometry (VBM) [11,14–17] and cortical thickness analysis [12] studies have demonstrated that cirrhotic patients without OHE exhibit abnormal volume, density and thickness of the gray matter (GM) in multiple brain regions, which indicates that the brain morphological alterations in those patients are widespread. Moreover, evidence from diffusion tensor imaging [13] has exposed disturbed structural connectivity within the default-mode network. These findings may lead investigators to posit that cirrhotic patients without OHE suffer alterations in the large-scale structural brain network.

Recent graph theoretical analyses offer a unique framework in which to quantify the topological and organizational properties of brain networks. Coordinated variations in brain morphology have been proposed as a valid measure to infer large-scale structural brain networks [18]. The structural networks constructed using morphometric correlations of GM volume or cortical thickness are consistent with those constructed from tract-tracing data and reflect the precise coordination of cortical morphology in the brain [18–21]. The small-world network, which is characterized by a high degree of clustering and short path lengths between individual network nodes, is a useful model for the description of complex brain networks [22]. Graph theoretical analyses have consistently demonstrated that brain structural networks in healthy individuals exhibit and allow for more efficient information processing than random networks [18,20,23]. To date, graph theoretical studies have revealed alterations of arrangements in structural correlation networks that are associated with normal aging [20], Alzheimer's disease [24], schizophrenia [21] and major depressive disorder [25]. Thus, changes identified in the structural network of the cirrhotic brain may enrich our understanding of the neuropathological mechanisms of this disease. So far, however, no such study has been published.

In the present study, we applied graph theoretical analysis to compare MRI-based GM volume correlation networks between healthy controls and cirrhotic patients. We specifically focused on investigating the changes in the brains of cirrhotic patients at the stage prior to OHE. Moreover, the cirrhotic patients included in this study were all HBV-RC subjects. This cirrhosis model should help to rule out the effects of certain confounding factors that can disrupt the central nervous system (e.g., alcohol and medications), which are common and significant in other types of cirrhosis models [26]. Given the previous evidence of a diffuse distribution pattern of GM structural abnormalities in cirrhotic patients, combined with recent findings of reductions in small-world characteristics and increased disorganization of functional networks in cirrhotic individuals [10], we hypothesized that topological alterations could be reflected in the brain structural correlation network. We also examined the between-group differences in regional network measures (betweenness centrality degreeand the distribution of hubs) to further highlight the most closely disease-related brain regions.

## Materials and Methods

### Subjects

The study was approved by the Research Ethics Review Board of Nanfang Hospital, Southern Medical University. Written informed consent was obtained from all participants before the study. Twenty-eight patients with HBV-RC without OHE (mean age: 45.75 ± 10.47 years, age range: 27–67; 25 males) and thirty normal controls (mean age: 44.37 ± 7.85 years, age range: 30–61; 23 males) matched for age, gender, and education were included.

In this study, we performed detailed and systematic examinations of each subject to establish a diagnosis of HBV-RC without OHE. In brief, HBV-RC was diagnosed through biopsy or on the basis of case history, clinical examination, and biochemical and imaging findings. Each patient’s liver functional status was assessed using the Child–Pugh score.

Patients were excluded for other types of viral hepatitis, OHE, gastrointestinal hemorrhage or bacterial infection (within 1 month before the study), a transjugular intrahepatic portosystemic or surgical portocaval shunt, and diffuse hepatocellular carcinoma. The exclusion criteria for all patients and controls also included age < 18 or > 70 years, alcoholism, neurological or psychiatric diseases, a history of substantial head trauma, infection with human immunodeficiency virus, diabetes, hypertension, poor vision, other major medical illness, left-handedness, and any focal abnormality detected upon routine brain MRI examination.

The clinical and demographic data from the patients with HBV-RC without OHE and the control subjects are summarized in Table 1.

**Table 1:** Demographic and clinical characteristics of participants.

Characteristic	HBV-RC group (n = 28)	CON group (n = 30)	*p*-value
Age (years) *	45.75 ± 10.47	44.37 ± 7.85	0.570
Sex ratio, M/F	25/3	23/7	0.301
Education (years) *	10.32 ± 3.74	11.63 ± 3.03	0.147
Child–Pugh A/B/C	15/8/5	-	-
Total serum bilirubin (IU/L) *	88.38±172.47	N/A	-
Serum albumin (g/L) *	35.87±6.15	N/A	-
Prothrombin time (s) *	16.67±2.77	N/A	-
PHES **	−2.5(−11∼1)	0(−3∼3)	<0.001

*Indicates means ± standard deviations.

**Indicates median and range.

Abbreviations:CON = control; HBV-RC = hepatitis B virus-related cirrhosis; N/A = not applicable; PHES = psychometric hepatic encephalopathy score.

### Neuropsychological Tests

The psychometric hepatic encephalopathy score (PHES) battery, which assesses multiple cognitive domains in patients with cirrhosis, including visual perception, construction, visual/spatial orientation, motor speed and accuracy, concentration, and attention [27], was administered to all 58 subjects to assess their cognitive function. In the present study, a previously reported version of the PHES battery was applied. The assessment comprised five paper-and-pencil psychometric tests, including the number connection test A (NCT-A, in seconds), the modified number connection test B (NCT-B, in seconds), the digit symbol test (DST, in numbers), the serial dotting test (SDT, in seconds) and the line-tracing test (LTT, the sum of time taken in seconds and the number of errors). All subjects completed the test battery after an appropriate explanation and demonstration. The method for calculating PHES values has been reported in detail elsewhere [6]. In brief, five formulae constructed using data from 133 healthy subjects in a previous study [6] were used to predict the expected results of the five neuropsychological tests. A recorded result of ≥ 1 standard deviation (SD) above the predicted value was scored as +1, results of 1 SD and 2 SDs below the predicted value were scored as −1 and −2, respectively, and a result equal to or below −3 SDs was scored as −3. The final PHES values were calculated as the sum of the scores from the five tests and ranged between +5 and −15.

### MRI Data Acquisition

MR imaging data were acquired for all subjects using a 1.5-T MR scanner (Achieva Nova-Dual, Philips Medical Systems, Best, Netherlands). Each subject lay in the supine position with the head snugly fixed with foam pads and a belt. The routine acquisition of T_1_-weighted and T_2_-FLAIR images was performed for each subject to detect clinically silent lesions in the central nervous system. High-resolution three-dimensional T_1_-weighted (3D-T_1_) images covering the entire brain were acquired in the sagittal orientation using a fast field echo (FFE) sequence (S1 Data). The imaging parameters were as follows: repetition time = 25 ms, echo time = 4.1 ms, flip angle = 30°, field of view = 230 × 230 mm, matrix = 231 × 231, slice thickness = 1 mm, and number of slices = 160.

### Structural Data Preprocessing

All 3D-T_1_ images were processed and examined using SPM8 software (http://www.fil.ion.ucl.ac.uk/spm). VBM was implemented in the VBM8 toolbox (http://dbm.neuro.uni-jena.de/vbm.html) with default parameters and with the incorporation of the DARTEL toolbox, which was used to obtain a high-dimensional normalization protocol [28]. The images were bias-corrected, tissue-classified, and registered using linear (12-parameter affine) and nonlinear transformations (warping) within a unified model. Subsequently, the warped GM segments were affine-transformed into Montreal Neurological Institute (MNI) space and were scaled using the Jacobian determinants of the deformations to account for the local compression and stretching that arose as a consequence of the warping and affine transformation (modulated GM volumes, S2 Data). The modulated GM images were further smoothed with an 8-mm full-width at half maximum (FWHM) isotropic Gaussian kernel. Then, the average GM volume of each brain region of interest (ROI) within the entire cerebrum was calculated using the WFU PickAtlas Toolbox implemented in SPM8 (S1 Table). Brain parcellation was performed following the automated anatomical labeling (AAL) algorithm [29] with 90 cortical and subcortical ROIs.

### Network Construction

A 90 × 90 association matrix *R* was constructed for each group using pair-wise regional correlations calculated as the partial correlation coefficients (*r*_*ij*_) between the average GM volume values for a pair of ROIs, “*i*” and “*j*”, in each group, with the subjects’ total brain volume and age included as covariates of nuisance. Thereafter, the matrix *R* was transformed into a binary matrix *A* by thresholding the *r*_*ij*_ into values of 0 or 1. Here, these thresholds were defined as a range of network densities (D_min_–D_max_), where D_min_ represents the minimum density above which neither network is fragmented [30]. In this study, we preliminarily calculated the network metrics across a density range of 0.1–0.45 (interval of density, 0.01) because structural networks with > 45% connectivity are considered to be less biologically meaningful [30]. Ultimately, the density region of interest for the global property comparisons was set to 0.20–0.40 because above 0.20, the networks of the HBV and control groups were fully connected (i.e., each node of the network had at least one connection with another node), and above 0.40, the small-worldness of both groups was < 1.2 and the networks were increasingly random [30]. For regional property comparisons, we selected a range of 0.20–0.33, throughout which the networks of the two groups were fully connected and exhibited between-group difference in small-worldness. Additionally, when regarding the matrix *A* as a graph *G*, we defined the quantities *N*, *K*, D, *i*, *j*, and *k* as the total number of ROIs (i.e., 90), the number of edges, the percentage of surviving edges at a given density threshold, and three randomly chosen nodes, respectively [31].

### Global Network Properties

For our constructed brain structural networks, we adopted a previously established graph theoretical analysis method to quantify the network properties [18]. This technique was developed based on the quantitative method for small-world architecture proposed by Watts and Strogatz [22]. The two basic measures are the characteristic path length *L* and the clustering coefficient *C*. Here, for a given graph *G* and two random nodes *i* & *j*, *L* is defined as the average of the shortest path lengths between two generic nodes:

$$L=\frac{1}{N\left(N-1\right)}\sum_{i\neq j\in G}L_{ij}$$

where *L*_*ij*_ is the length of the shortest path between *i* and *j*. Therefore, *L* essentially reflects the degree of network integration and the capacity of a network to process information at the global level [32].

The clustering coefficient of a node *i* (*C*_*i*_) measures the number of edges among its nearest neighbors, and *C* is correspondingly defined as the average of the local clustering coefficients *C*_*i*_ of all nodes:

$$C_{i}=\frac{1}{N(N_{G_{i}}-1)}\sum_{j,k\in N_{G_{i}}}1/L_{jk},C=\frac{1}{N}\sum_{i\in G}C_{i}$$

where *G*_*i*_ is a subgraph from the whole network composed of the nearest neighbors of node *i*. Thus, *C* is a measure of network segregation reflecting the efficiency of a network in processing information locally. To better summarize the topology of the brain network, we further calculated the normalized path length *λ* and the normalized clustering coefficient *γ* by comparing them with the random network [22,23,33]:

$$\lambda =L/L_{\mathrm{rand}},\gamma =C/C_{\mathrm{rand}}$$

where *L*_*rand*_ and *C*_*rand*_ are the means of the characteristic path lengths and the clustering coefficients, respectively, of 1000 randomly generated networks matched for nodes, edges, and degree distribution.

Finally, based on *λ* and *γ*, the small-worldness index *σ* of the brain network was calculated as follows:

σ=γ/λ

which is a more direct metric for the small-world organization of the brain network.

### Regional Network Properties & Hub Identification

In this study, the regional network properties were summarized and quantified using the normalized betweenness centrality *b*_*i*_ and the normalized degree *k*_*i*_:

$$b_{i}=B_{i}/\bar{B},k_{i}=K_{i}/\bar{K}$$

where *B*_*i*_ represents the total number of shortest paths passing through node *i* [34], *K*_*i*_ represents the number of edges between node *i* and other nodes, and $\bar{B}$ and $\bar{K}$ represent the mean betweenness and degree, respectively, of the entire network. Furthermore, *b*_*i*_ and *k*_*i*_ were used to identify the hubs of the network, i.e., nodes with regional values at least 1.5 SDs greater than the mean value were recognized as hubs [35].

### Statistical Analysis

#### Demographic and Cognitive Testing

Two-sample t-tests were performed to assess the differences in age and duration of education between the cirrhotic patients and the healthy subjects. Pearson’s chi-squared test was used to compare gender ratios between the two groups. The Mann-Whitney U test was used to analyze the differences in PHES test performance between the two groups. These analyses were performed using commercially available statistical software (SPSS, version 13.0; Chicago, IL, USA), and a *p*-value of less than 0.05 (two-tailed) was deemed significant.

### Between-Group Network Comparisons

Between-group comparisons with respect to global (*λ*, *γ* and *σ*) and regional (*b*_*i*_ and *k*_*i*_) network properties were performed using the nonparametric permutation test [24,36,37]. The Graph Analysis Toolbox [37] was used for the between-group analyses, and a *p*-value of 0.05 (two-tailed) was considered to be statistically significant. The global network properties were compared across a density range of 0.20–0.40. For the regional measures *b*_*i*_ and *k*_*i*_, the between-group differences were compared using an area under the curve (AUC) analysis of the networks over a density range of 0.20–0.33; in this range, the two groups exhibited fully connected networks and a significant difference in small-worldness. The statistical threshold was initially set to *p* < 0.05 (uncorrected) and further to *p* < 0.001 (uncorrected).

## Results

### Demographic and Cognitive Testing

There were no significant differences in age, sex or education between the cirrhotic patients and the control subjects. Compared with the control subjects, the cirrhotic patients had significantly lower PHES scores (−2.5 [−11 to 1] vs. 0 [−3 to 3], respectively; *p* < 0.001) (Table 1).

### Changes in Global Network Properties

**Fig 1:**
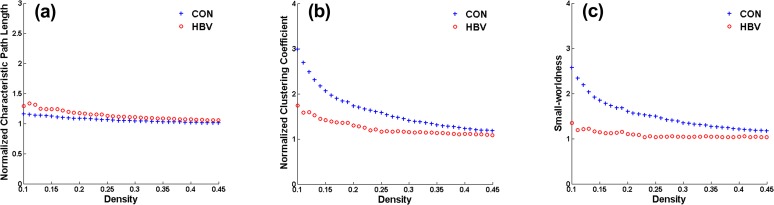
Changes in global network measures as a function of network density. Normalized characteristic path lengths **(a)**, normalized clustering coefficients **(b)**, and small-world indices **(c)** of the network of the hepatitis B virus-related cirrhosis (HBV-RC) group and the control (CON) group. The network properties are calculated across a density range of 0.10–0.45 (density interval, 0.01).

**Fig 2:**
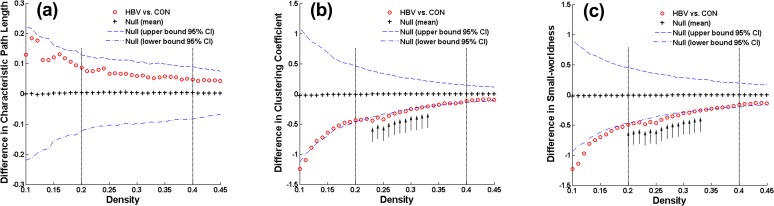
Between-group differences in global network measures between the hepatitis B virus-related cirrhosis (HBV-RC) group and the control (CON) group as a function of network density. The graph shows that there was no significant difference in the network characteristic path length **(a)** between the groups, whereas the clustering coefficient **(b)** was significantly decreased in the HBV-RC network at various densities, resulting in lower small-worldness **(c)** compared with the CON group across a density range of 0.20–0.40 (between the vertical dotted lines). The vertical arrows represent the densities at which the difference was statistically significant at *p* < 0.05. The line corresponding to the ○symbols represents the CON group measure minus the HBV-RC group measure, the line corresponding to the + symbols represents the mean of the random-graph distribution, and the dashed lines indicate the 95% confidence interval.

The density range of interest for global properties was ultimately set to 0.20–0.40; in this range, the networks of the HBV group and the control group were not fragmented and were of biological significance. Throughout this density range, the control group exhibited efficient small-world topology, i.e., *λ* ≈ 1, *γ* > 1, and *σ* > 1.2, whereas the HBV-RC network presented an altered topological organization, i.e., a tendency toward increased *λ* and decreased *γ* and *σ* (Fig. 1). Statistically, the small increase in *λ* in the HBV group was non-significant (Fig. 2A), whereas *γ* was significantly decreased at densities of 0.23–0.33 (Fig. 2B), resulting in significantly decreased *σ* between 0.20 and 0.33 (Fig. 2C).

### Regional Comparison & Hubs

**Fig 3:**
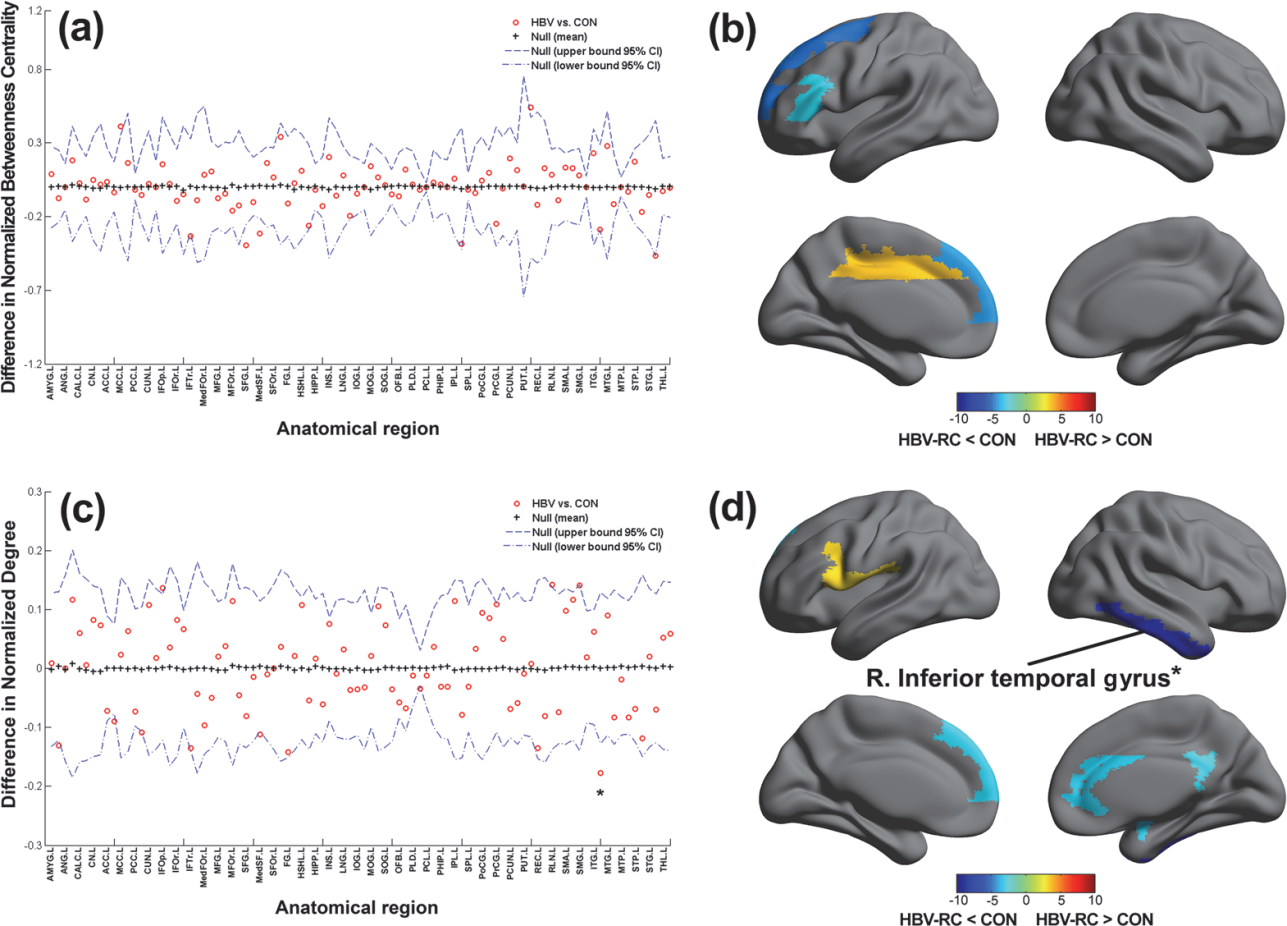
Between-group differences in regional network topology. (**a-b)** The hepatitis B virus-related cirrhosis (HBV-RC) group exhibited decreased network betweenness centrality in the left inferior frontal gyrus (triangular part), the superior frontal gyrus, and the medial superior frontal gyrus, and exhibited increased network betweenness centrality in the left middle cingulum and the right putamen. **(c-d)** The HBV-RC group exhibited a decreased network degree in the right amygdala, the anterior cingulum, the post cingulum, the pallidum, the inferior temporal gyrus, and the left medial superior frontal gyrus, and exhibited an increased network degree in the left inferior frontal gyrus (opercular part) and the left rolandic operculum. *indicates *p* < 0.001. Abbreviations: L = left hemisphere; R = right hemisphere; AMYG = amygdala; ANG = angular gyrus; CALC = calcarine fissure; CN = caudate nucleus; ACC = anterior cingulate; MCC = mid-cingulate; PCC = posterior cingulate; CUN = cuneus; IFOp = inferior frontal gyrus, opercular part; IFOr = inferior frontal gyrus, orbital part; IFTr = inferior frontal gyrus, triangular part; MedFOr = medial frontal gyrus, orbital part; MFG = middle frontal gyrus; MFOr = middle frontal gyrus, orbital part; SFG = superior frontal gyrus; MedSF = superior frontal gyrus, medial part; SFOr = superior frontal gyrus, orbital part; FG = fusiform gyrus; HSHL = heschl gyrus; HIPP = hippocampus; INS = insula; LNG = lingual gyrus; IOG = inferior occipital gyrus; MOG = middle occipital gyrus; SOG = superior occipital gyrus; OFB = olfactory cortex; PLD = lenticular nucleus, pallidum; PCL = paracentral lobule; PHIP = parahippocampal gyrus; IPL = inferior parietal lobule; SPL = superior parietal lobule; PoCG = postcentral gyrus; PrCG = precentral gyrus; PCUN = precuneus; PUT = putamen; REC = gyrus rectus; RLN = rolandic operculum; SMA = supplementary motor area; SMG = supramarginal gyrus; ITG = inferior temporal gyrus; MTG = middle temporal gyrus; MTP = middle temporal pole; STP = superior temporal pole; STG = superior temporal gyrus; THL = thalamus.

AUC analysis of the regional network properties revealed a series of brain regions with different metrics (Table 2). Compared with the control group, the *b*_*i*_ values in the patients with HBV-RC were decreased (*p* < 0.05, uncorrected) in the left inferior frontal gyrus (triangular part), the superior frontal gyrus, and the medial superior frontal gyrus and were increased in the left middle cingulum and the right putamen (Fig. 3A and 3B). A decrease in *k*_*i*_ was observed in the right amygdala, the anterior cingulum, the postcingulum, the pallidum, the inferior temporalgyrus, and the left medial superior frontal gyrus, and this value was increased in the left inferior frontal gyrus (opercular part) and the left rolandic operculum (Fig. 3C and 3D). After the threshold was increased to *p* = 0.001, the right inferior temporal gyrus persisted with a significantly decreased *k*_*i*_ (Fig. 3D). Together, these regions were predominantly distributed throughout the association cortices (frontal and temporal regions), the paralimbic cortices (cingulum andamygdala), and one subcortical region (pallidum).

**Table 2:** Comparison of regional network properties between patients and normal controls based on AUC analysis.

Region	Class	Direction	*p*
**Betweenness centrality**			
L.Frontal_Inf_Tri	Association	↓	0.048
L.Frontal_Sup	Association	↓	0.007
L.Frontal_Sup_Med	Association	↓	0.014
L.Cingulum_Mid	Paralimbic	↑	0.027
R.Putamen	Subcortical	↑	0.033
**Degree**			
R.Amygdala	Paralimbic	↓	0.045
R.Cingulum_Ant	Paralimbic	↓	0.039
R.Cingulum_Post	Paralimbic	↓	0.049
L.Frontal_Sup_Med	Association	↓	0.043
R.Pallidum	Subcortical	↓	0.043
R.Temporal_Inf	Association	↓	**0.001** *
L.Frontal_Inf_Oper	Association	↑	0.040
L.Rolandic_Oper	Association	↑	0.047

↑ indicates that the metric was higher in the patients with hepatitis B virus-related cirrhosis;

↓ indicates that the metric was higher in the controls;

* indicates that the region was statistically significant after the significant *p*-value was adjusted to 0.001. The regions were classified as primary, association, and paralimbic as described by Mesulam (1998).

Abbreviations: L = left; R = right; Sup = superior; Inf = inferior; Med = medial; Orb = Orbital; Oper = Opercular; Tri = triangular; Ant = anterior; Post = posterior.

**Fig 4:**
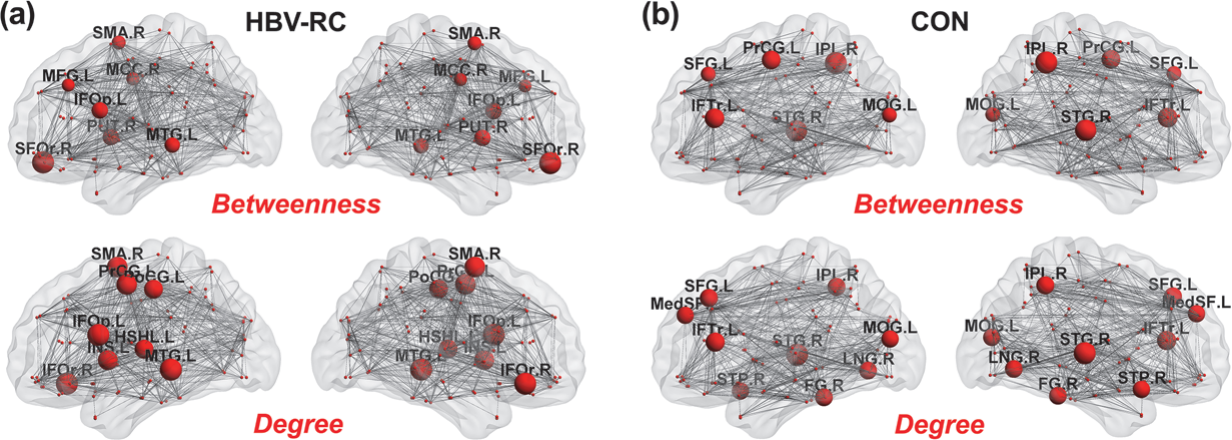
Network hubs. Hubs of the networks constructed for the hepatitis B virus-related cirrhosis (HBV-RC) group **(a)** and the control (CON) group **(b)** based on nodal betweenness and nodal degree. The gray lines indicate connections (edges), and the spheres represent regions (nodes). The hubs are magnified, with radii proportional to the nodal metrics. The hubs are marked in red. Abbreviations: L = left hemisphere; R = right hemisphere; MCC = mid-cingulate; IFOp = inferior frontal gyrus, opercular part; IFOr = inferior frontal gyrus, orbital part; IFTr = inferior frontal gyrus, triangular part; MFG = middle frontal gyrus; SFG = superior frontal gyrus; MedSF = superior frontal gyrus, medial part; SFOr = superior frontal gyrus, orbital part; FG = fusiform gyrus; HSHL = heschl gyrus; INS = insula; LNG = lingual gyrus; MOG = middle occipital gyrus; IPL = inferior parietal lobule; PoCG = postcentral gyrus; PrCG = precentral gyrus; PUT = putamen; SMA = supplementary motor area; MTG = middle temporal gyrus; STP = superior temporal pole; STG = superior temporal gyrus.

The names of the hubs in the networks constructed for the patient (HBV-RC) and control (CON) groups are listed in Table 3 and Fig. 4. Ten network hubs were identified in the CON group based on *k*_*i*_ and *b*_*i*_, located predominantly in the association cortices (8/10), the paralimbic cortex (1/10) and the primary cortex (1/10), whereas twelve hubs were detected in the HBV-RC group, located predominantly in the association cortex (6/12), the primary cortex (3/12), the paralimbic cortex (2/12) and the subcortical cortex (1/12). Altogether, compared with the CON group, more hub regions were identified in the HBV-RC group, indicating a loss of association hubs and the emergence of an increased number of non-association hubs.

**Table 3:** Hubs in patients and normal controls, as defined based on normalized betweenness centrality and degree.

**HBV-RC**				**CON**			
**Region**	**Class**	**Average *b*_*i*_/*k*_*i*_**		**Region**	**Class**	**Average *b*_*i*_/*k*_*i*_**	
		***b*_*i*_**	***k*_*i*_**			***b*_*i*_**	***k*_*i*_**
L.Frontal_Inf_Oper	Association	5.987*	9.100*	L.Frontal_Inf_Tri	Association	7.752*	7.463*
R.Frontal_Inf_Orb	Association	3.295	9.100*	L.Frontal_Sup	Association	4.997*	6.850*
R.Frontal_Sup_Orb	Association	9.100*	2.839	L.Frontal_Sup_Med	Association	4.463	6.440*
L.Frontal_Mid	Association	3.969	7.143*	R.Parietal_Inf	Association	9.100*	6.850*
R.Supp_Motor_Area	Association	4.494*	8.317*	R.Temporal_Sup	Association	8.749*	9.100*
L.Temporal_Mid	Association	5.315*	9.100*	R.Fusiform	Association	4.272	6.645*
R.Cingulum_Mid	Paralimbic	4.062	6.556*	R.Lingual	Association	4.709	6.645*
L.Insula	Paralimbic	3.369	7.926*	L.Occipital_Mid	Association	4.959*	6.440*
L.Precentral	Primary	1.771	8.317*	R.Temporal_Pole_Sup	Paralimbic	4.084	6.440*
L.Postcentral	Primary	2.395*	7.339	L.Precentral	Primary	**7.260** *	5.213
L.Heschl	Primary	2.480	7.339*				
R.Putamen	Subcortical	5.875*	1.273				

* indicates nodes with regional values at least 1.5 standard deviations greater than the mean value. The regions were classified as primary, association, and paralimbic as described by Mesulam (1998).

Abbreviations: CON = control; HBV-RC = hepatitis B virus-related cirrhosis; L = left; R = right; Sup = superior; Inf = inferior; Med = medial; Mid = middle; Orb = Orbital; Oper = Opercular; Tri = triangular.

## Discussion

This is the first study to use graph analysis to investigate alterations in the GM volume correlation networks of patients with HBV-RC without OHE. Our main findings are as follows: (1) the global topological organization of the GM volume networks in cirrhotic patients was disrupted, as indicated by altered small-world parameters; (2) brain areas with altered nodal degree/betweenness centrality were observed predominantly in association cortices (frontal and temporal regions) in cirrhotic patients; (3) different patterns in hub distribution were observed in the cirrhotic patients versus the controls based on nodal betweenness and nodal degree. Taken together, our findings indicate that the topological organization of the structural networks constructed based on GM volumes in patients with HBV-RC without OHE is less optimal than that of the networks corresponding to healthy controls, indicating a more limited capability to integrate information among the different regions of the brain.

### Disrupted Global Topological Organization

In this study, the structural network of the control group was found to exhibit efficient small-world organization, consistent with previous functional and structural studies [18,25,37]. The HBV-RC group network, however, exhibited significantly altered small-world indices (i.e., decreased clustering coefficient and decreased small-worldness) compared with the control group.

The observed decrease in the clustering coefficient, which measures network segregation at the global level, may reflect a relatively sparse local connectedness of the brain structural networks in patients with HBV-RC. These findings are consistent with a number of recent resting-state functional studies [8,10]. Zhang et al. [8] have observed the widespread occurrence of weaker cortical and subcortical network connectivity in correlationwith neuropsychological impairment in cirrhotic patients with minimal HE. Hsu et al. [10] have also observed that global topological efficiencies of the functional connectivity network were significantly disrupted in cirrhotic patients. Moreover, the results of a network-level analysis corroborate previous structural neuroimaging findings that have demonstrated a widespread pattern of atrophy in GM volumes in cirrhotic patients [11], which may further suggest an alteration in the coordinated patterns of brain morphology in the structural networks of these patients.

Taken together, our findings suggest that the structural correlation networks of patients with HBV-RC tend to exhibit more randomized configurations compared with those of normal individuals. Given that the small-world model reflects an optimal balance between local specialization and global integration, as described previously [22,23], the decreased clustering coefficient thus indicates a shift toward random networking caused by decreased local segregation. Therefore, the altered small-world parameters in structural networks may reflect a less optimal topological organization in patients with HBV-RC without OHE.

### Between-Group Differences in Regional Network Measures

In this study, we performed regional network comparisons using normalized betweenness centrality and normalized degree to help localize abnormal brain areas in HBV-RC patients. With a relatively liberal statistical threshold (*p* < 0.05, uncorrected), we observed both decreased and increased regional nodal degree/betweenness in multiple brain regions in the HBV-RC group, predominantly located in the association cortices (frontal and temporal regions), the paralimbic cortices (cingulum and amygdala), and one subcortical region (pallidum). A recent VBM study using MRI [11] has indicated that the decreased GM volume detected in cirrhotic patients with minimal HE is predominantly located in the frontal and temporal cortices, the paracentral lobule, the caudate, the putamen and the amygdala. Pathologically, Alzheimer type II astrocytes in the GM and a diffuse pseudolaminarspongy degeneration of the cortex, the putamen, the pallidum, and the caudate nucleus are characteristic findings in cirrhotic patients with chronic HE [38]. Taken together, these results indicate that such diffuse GM structural abnormalities may be at least partially responsible for the widespread regional changes observed in the network properties.

After the threshold was increased to *p* = 0.001, a decreased regional nodal degree in the inferior temporal gyrus was observed. As part of the ventral pathway, the inferior temporal gyrus is important to high-level visual processing [39]. Neuroimaging studies [6–9] have consistently found that patients with cirrhosis display abnormal resting-state brain activity and functional connectivity within the visual-association areas. Thus, our finding of a decreased regional nodal degree in visual-association areas provides new evidence that the visual-associated subnetwork is relatively vulnerable to neurological impairments caused by HBV-RC.

### Network Hub Analysis

As crucial components required for efficient communication in a network, hubs regulate information flow and play a key role in network resilience against insult. In the present study, the number (6/12 vs. 8/10) and the distribution of association hubs varied between the two groups. Among the abnormal changes in the hub regions observed in the HBV-RC group, the inferior parietal gyrus belongs to the default mode network, which has been hypothesized to be profoundly relevant to various cognitive processes, such as visual attention, memory, and motor activity [40]. The fusiform, the lingual gyrus and the middle occipital gyrus are intimately involved in visual information processing [41,42]. The fact that the network of the HBV-RC patients did not exhibit the expected association hubs suggests the occurrence of network-level changes in these regions, which may be functionally linked to the visual association abilities, motor-related performance, attention and memory function of the HBV-RC patients assessed in this study.

Interestingly, the total number of hubs increased in the HBV-RC group, with the addition of non-association hubs (paralimbic, primary and subcortical regions).These findings are consistent with a recent study of the functional connectivity networks of cirrhotic patients without OHE [43], which also observed an increased total number of hubs. These findings may be explained in terms of compensation for the disrupted modularity in non-HE patients [43]. Thus, the increased number of non-association hubs observed in our study may be a compensatory mechanism for the decreased number of hubs and the overall hub disruption in the association cortex. This compensatory mechanism may enable HBV-RC patients to use additional resources to approach a normal level of cognition.

### Limitations

This study has several limitations. First, the sample size included in this study was relatively small. In addition, although the image quality was rigidly controlled, we used a 1.5T-MR scanner; better WM-GM resolution may be achieved in future work using a 3.0T-scanner. Second, because a proper cutoff PHES for the diagnosis of minimal HE has not yet been established in our country, further group analysis is needed in future studies. Third, because of the group-wise nature of the method used to calculate the structural networks, no correlation analysis between network properties and cognitive measurements (PHES) could be performed to further specify the causal relationship between HBV-RC and network alterations. Fourth, the nodal degree/betweenness centrality results did not persist when we performed multiple comparisons, meaning that this study should be regarded as an exploratory analysis. For increased statistical power, the findings should be replicated using a larger sample of subjects. Finally, our understanding of the basic biology behind the aberrations in the structural network properties of HBV-RC patients is still very limited. Previous GM structural studies [11,12,14–17] related to the cirrhotic brain have primarily focused on morphometry (such as volume and thickness), which are different metrics from those used in the construction of structural networks. Thus, it may be not reasonable to discuss the consistency between our study and these previous studies, and the explanation of the results provided in this study should be treated with caution.

## Conclusions

Despite these limitations, the present findings enhance our understanding of the neural basis of the cognitive deficits observed in patients with HBV-RC without OHE by demonstrating aberrations in the network properties of the cirrhotic brain, specifically the decreased clustering coefficient and the decreased small-worldness, which suggest the reduced efficiency of the network. The observed regional pattern of abnormalities in the association cortices (frontal and temporal regions) may be related to the impaired cognitive abilities of cirrhotic patients. These findings provide new insight into the interregional reorganization occurring in the brains of these patients from a network perspective.

## Supporting Information

S1 Data Raw 3D-T1WI image of subject No.1. (GZ)

S2 Data Warped grey matter image of subject No.1 in Montreal Neurological Institute space using the DARTEL toolbox. (GZ)

S1 Table Extracted average grey matter volume of 90 brain regions from each subject. (XLSX)
